# Anatomical and Functional Alterations in Nasolacrimal Duct Obstruction: A Comprehensive Review

**DOI:** 10.7759/cureus.90754

**Published:** 2025-08-22

**Authors:** David Sáenz Araya, Freddy Lizano Guevara, Enmanuel Sevilla Torres, Daniela Fernandez Vinocour, Alberto Rojas Peláez

**Affiliations:** 1 General Medicine, Universidad de Ciencias Médicas (UCIMED), San José, CRI

**Keywords:** congenital abnormalities, dacryocystorhinostomy, epiphora, lacrimal apparatus, lacrimal gland, nasolacrimal duct obstruction, tear film

## Abstract

The lacrimal drainage system, or LDS, is an intricate anatomical and functional system that mobilizes and drains tears from the ocular surface into the nasal cavity. The drainage system first begins at the puncta, small openings located at the medial eyelids which actively funnel tears into the canaliculi. The canaliculi drain into the lacrimal sac (LS), with the LS residing in the lacrimal fossa, which collects the tear fluid and subsequently drains the fluid into the nasolacrimal duct (NLD), which empties into the inferior meatus of the nasal cavity. Variations in canalicular length and duct curvature and diameters influence the dynamics of tear flow in the LDS as well as the potential for obstruction. It should be noted that females appear to have narrower NLDs, which could contribute to the higher rates observed for nasolacrimal duct obstruction (NLDO).

Importantly, the lacrimal pump mechanism activates during blinking by the Horner-Duverney part of the orbicularis oculi muscle on each side. There are valves in the LDS, like the valve of Rosenmüller, that allow unidirectional flow and prevent anterograde flow from the NLD into the inferior meatus nasal or pharyngeal region. An obstruction to the flow of tears out of the lacrimal drainage system could cause secondary changes in the lacrimal gland (LG), such as altered protein secretion properties, histologic remodeling in the LG, or altered migration of immune cells. The inflammatory and fibrotic processes involved in the progression of NLDO are likely mediated by T and B lymphocytes and fibrogenic cytokines that create fibroblasts which leads to progressive narrowing of the ductal lumen by various inflammatory and fibrotic processes such as lap-time would reveal.

Anatomical influences related to NLDO include canalicular stenosis, dacryocystocele, deviated nasal septum (DNS), inferior turbinate hypertrophy (ITH), and sinus pathology. Diagnosis of NLDO is made through a clinical examination with imaging studies, including computed tomography (CT) or dacryocystography (DCG), and functional studies. Treatment includes conservative therapy, probing, balloon dacryoplasty, and dacryocystorhinostomy (DCR); outcomes are multifactorial, including etiology, age, and comorbidity.

## Introduction and background

Nasolacrimal duct obstruction (NLDO) is a common condition with varying implications based on age. In newborns, there is a large prevalence of CNLDO (estimated to be 11% to 20% of newborns) which can be disconcerting at first when diagnosed, but most patients resolve either spontaneously or with minimal effort such as massage and topical antibiotics over the first year of life. However, when obstruction persists, it can go beyond excessive tearing. Studies in animal models have shown that chronic NLDO is associated with ocular surface changes, including damage to the corneal epithelium and either reduced number or function of goblet cells critical to a stable tear film [1,2].

For adult populations, NLDO presents distinct considerations, often more successfully addressed surgically. Dacryocystorhinostomy (DCR) is commonly external or endoscopic and remains the common standard of care. DCR success depends on the type and degree and totality of obstruction, and for completely patent NLDO patients, higher rates of symptomatic improvement after endoscopic DCR usually occur compared to patients who have had the diagnosis of nasolacrimal duct stenosis (NLDS). As re-processes and surgical transitions progress and advances through an era of delicate surgical techniques, distinct procedures have also developed. For instance, dacryoendoscopic-assisted laser dacryoplasty with silicone intubation (DLDI) is one new and beneficial treatment model with the ability to be considered for selected limited cases of NLDO, while ultimately controlling variables of the extent of ductal obstruction, inflammation, and fibrosis affecting the lacrimal pathway [3,4].

For pediatric patients with an ongoing or anatomically complicated obstruction, unique anatomical congenital stenosis may benefit from new surgical interventions such as endoscopic endonasal procedures. The benefits of these minimally invasive surgical techniques are the appropriate visualization and re-anatomizing of congenital stenoses of the lacrimal system with an easier, potentially safer option, with a unique end in mind and refining a different procedure than traditional probing or external surgery case by case [1].

In addition to anatomic obstruction, there are genetic and structural elements that also contribute to the pathogenesis of NLDO. A specific, though rare, type is bony congenital NLDO (CNLDO), which has been associated with aplasia of lacrimal and salivary glands and is related to mutations in the Fgf10 gene [5]. Rare genetic conditions as bony congenital NLDO, which do not respond to conventional treatments, strongly recommend expanding the diagnostic approach in an assessment for cases of congenital obstruction that do not respond to conservative approaches. In addition, NLDO has also been shown to induce structural changes in the lacrimal gland, which can also alter the composition and secretion of tears. These changes show a bidirectional relationship within the lacrimal functional unit, whereby distal outflow obstruction may secondarily impair the secretory function of the lacrimal gland [2].

The objective of this review is to thoroughly evaluate the changes, both anatomical and functional, that occur with NLDO, in its congenital and acquired forms.

## Review

Methods

This narrative review presents a focused yet flexible synthesis of current literature to provide a comprehensive overview of the anatomical, physiological, and functional changes associated with NLDO, in both congenital and acquired forms. The review also incorporates recent developments in clinical management, pathophysiology, and relevant structural aspects of the lacrimal system.

A comprehensive literature search was performed using three major biomedical databases, PubMed, Scopus, and ScienceDirect, which provide broad access to peer-reviewed publications in medicine and life sciences. No date restrictions were imposed on the initial search; however, the final selection prioritized studies published between 2019 and 2025 to ensure clinical relevance and contemporary evidence. Articles were limited to those published in English or Spanish to ensure accurate interpretation and minimize misinterpretation of findings.

The search strategy utilized both Medical Subject Headings (MeSH) and free-text keywords, including terms such as: Nasolacrimal Duct Obstruction, Congenital Abnormalities, Dacryocystorhinostomy, Lacrimal Apparatus, Epiphora, Lacrimal Gland, Tear Film. Boolean operators were applied to optimize search precision and focus results on anatomical, clinical, and surgical insights.

Rather than adhering to rigid systematic review protocols, a flexible and context-sensitive approach was adopted for study selection, appropriate for a condition influenced by diverse variables. Studies were selected based on scientific relevance, methodological rigor, and their contribution to understanding NLDO in a clinically applicable manner. Only original research articles, systematic reviews, meta-analyses, and official guidelines or consensus statements from recognized professional bodies were considered. Redundant publications, non-clinically applicable articles, and studies lacking relevant outcome data were excluded.

The final selection included a variety of evidence types experimental studies, observational research, clinical reviews, and surgical case series allowing for a multidimensional analysis of NLDO. This broad methodological base supports an integrated interpretation of the condition, particularly regarding lacrimal anatomy, functional impairment, genetic correlations, and advancements in therapeutic strategies.

To improve clarity and coherence, organizational tools including artificial intelligence were used in the editing process. All content interpretation, critical analysis, and scientific decisions were performed by the authors, ensuring academic integrity, accuracy, and scholarly value of the review.

Anatomy of the lacrimal drainage system

**Figure 1:**
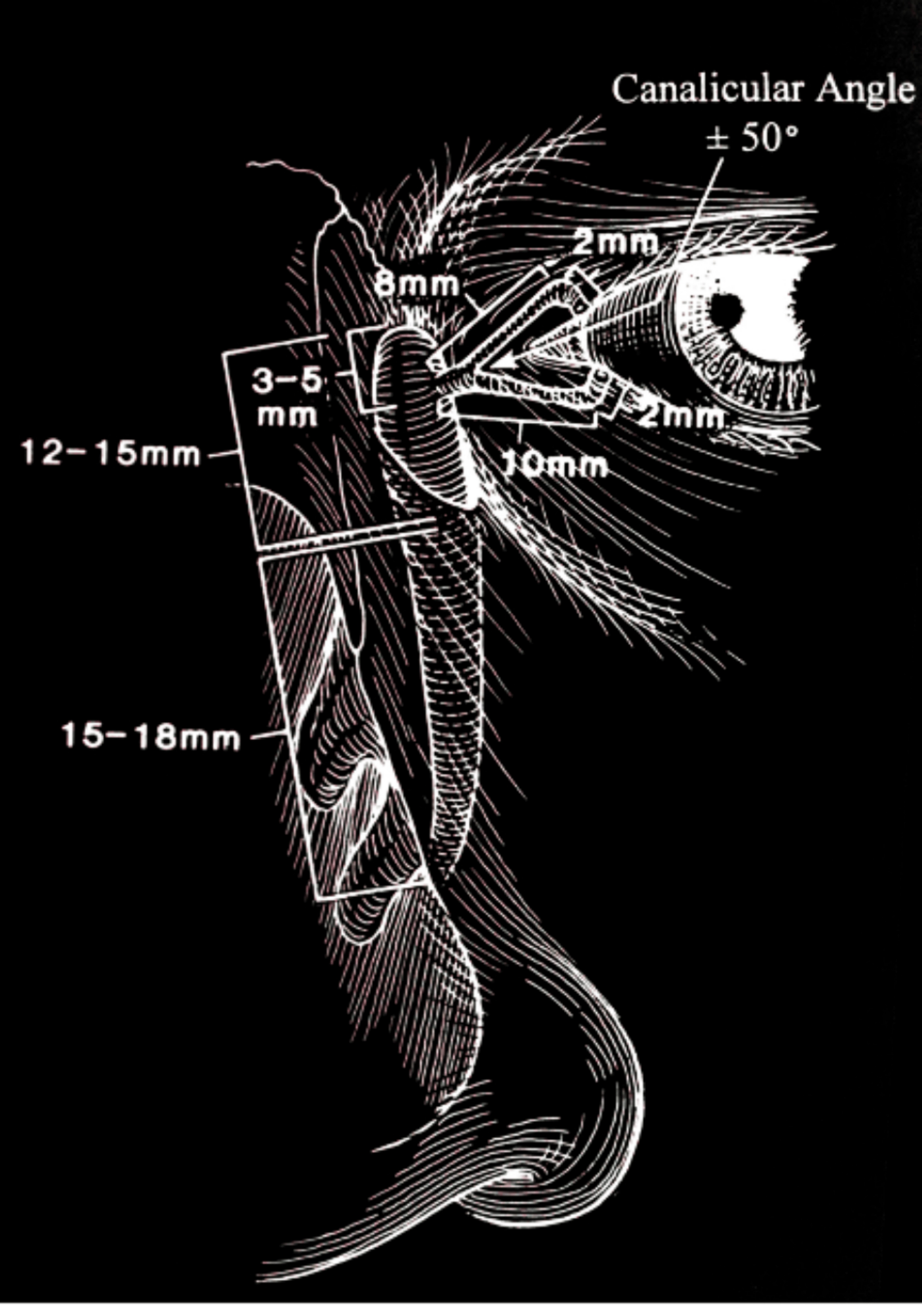
Schematic anatomy and dimensions of the adult lacrimal drainage system. Figure reproduced from Pezzoli et al., licensed under CC BY-NC-ND 4.0 [7].

The lacrimal drainage system is a complex anatomical and functional structure that directs tears from the ocular surface to the nasal cavity. This architecture contains interconnected components that provide uniquely coordinated functions for tear homeostasis. The system begins with tears entering the drainage system through small openings called puncta positioned at the medial canthus of each eyelid. These orifices are found at the opening to the canaliculi that transport tear fluid toward the lacrimal sac. The overall structure and spatial arrangement of these components are illustrated in Figure 1, which presents the schematic anatomy and dimensions of the adult lacrimal drainage system. Importantly, comparative anatomical studies on lacrimal drainage have demonstrated that the canaliculi of brachycephalic dog breeds are longer than their normocephalic counterparts, which likely has important implications for tear flow dynamics and efficiency [6].

The lacrimal sac is located within the lacrimal fossa and acts as the repository that collects tears from the canaliculi. The lacrimal sac is hidden quite deep in the orbit, near the base of the skull, and although during surgical intervention, such as DCR, there remains a very small possibility of complications such as a cerebrospinal fluid leak, this is not as significant due to the intervening presence of the frontal sinus between the base skull and lacrimal sac [8]. Tears from the lacrimal sac flow into the nasolacrimal duct (NLD), which drains into the inferior meatus of the nasal cavity. The NLD has considerable interindividual variability with respect to its length, curvature, and internal diameter, and both individual variants and variations in a particular individual can strongly influence the risk of obstruction. For instance, female patients have been noted to have narrower NLDs than males, which may account for the higher incidence of nasolacrimal duct obstruction found in female patients [9]. 

**Figure 2:**
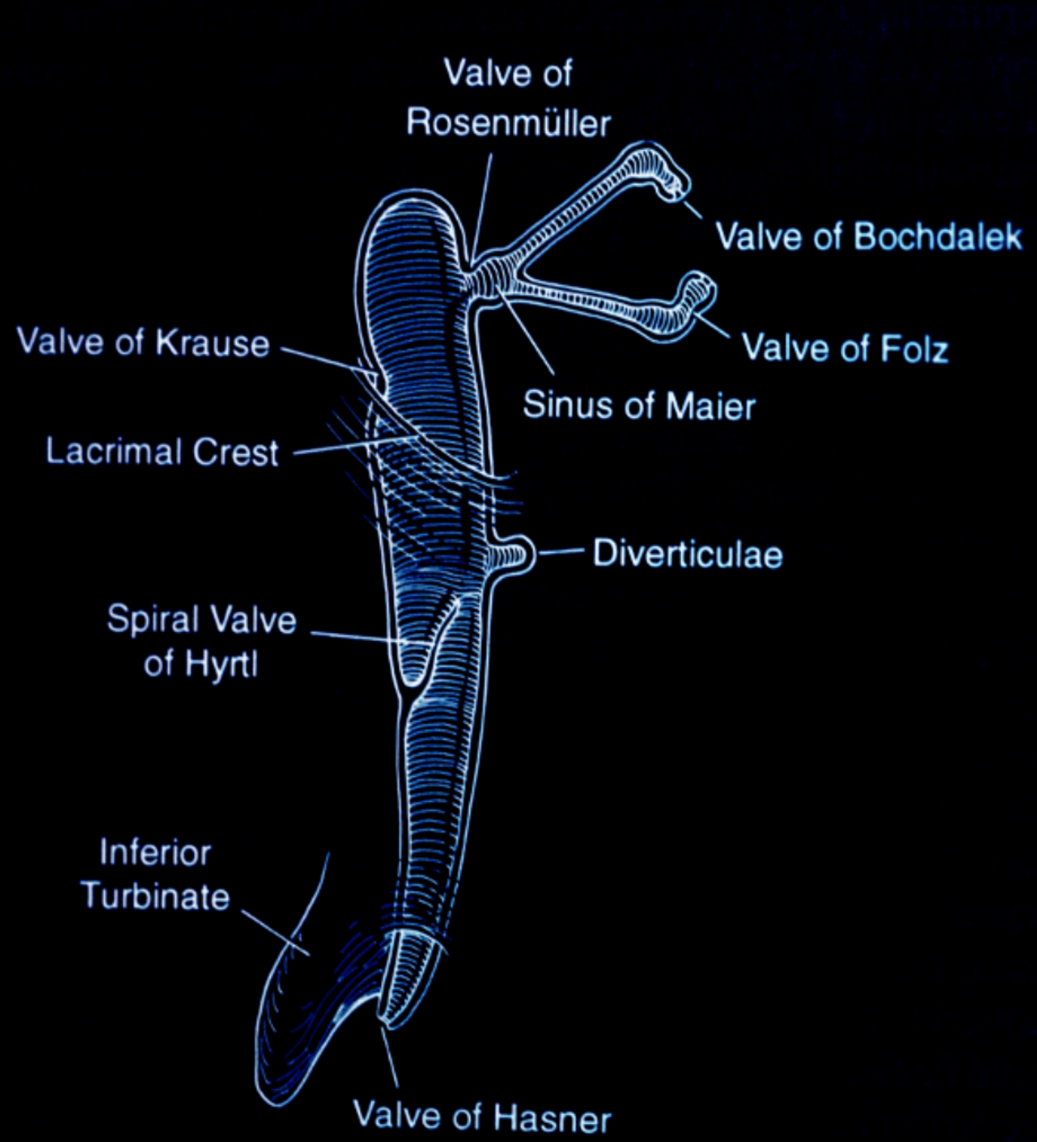
Anatomical valves and sinuses of the lacrimal drainage system. Figure reproduced from Pezzoli et al., licensed under CC BY-NC-ND 4.0 [7].

At the microscopic level, the lacrimal drainage system is lined by a mucosal layer with anatomical landmarks that correspond to its function. Of note is the valve of Rosenmüller, which plays an important role in maintaining the unidirectional flow of tears while minimizing the retrograde flow of nasal contents into the tear drainage system [10]. Figure 2 illustrates the location and configuration of these anatomical valves and surrounding sinuses, emphasizing their relevance in functional tear flow and protection against retrograde contamination [7].

Disruption of this mucosal continuity or mechanical failure of these valves may merely serve to predispose individuals to lagophthalmos and recurrent infections. In addition, histological studies have demonstrated that obstruction of the drainage system may also induce secondary changes to the lacrimal gland itself. For example, it has been reported that obstructed drainage results from the impaired fusion of secretory vesicles, indicating a functional collaboration or dependence of tears' production and drainage mechanism [2].

The lacrimal system also involves the use of a complicated vascular and neural network for the execution of its physiological impacts. Blood supply is mainly from the branches of the ophthalmic artery, and autonomic innervation - specifically parasympathetic fibers - regulates both lacrimation, and the tone of smooth muscle elements along the drain pathway. Both dependent systems have the potential to further worsen existing mechanical obstruction or disadvantage spontaneous compensatory mechanisms [11].

In clinical practice, anatomical variations are significant contributors to the causes of NLDO and its etiology. For example, a decreased minimum transverse diameter of the NLD is one variant that has been shown to be specific to primary acquired nasolacrimal duct obstruction (PANDO) - a condition commonly seen in middle-aged adult patients [12]. In addition, mucosal folds can also be found at the junction of the canaliculi and lacrimal sac which may mimic mechanical obstructions during examination, which may complicate an accurate diagnosis and resolution [13]. Brachycephalic dog breeds continue to be useful NLDO models due to their distinctive craniofacial and nasolacrimal duct morphology. Such animals are more likely to have steep duct angulations, and normally possess accessory nasolacrimal openings, which can complicate tear drainage as well as induce ocular discharge chronically [6].

Physiology of lacrimal drainage

The processes of tear production and drainage are closely interrelated, and changes in one are often associated with changes to the other. For example, in cases of NLDO, the lacrimal gland on the affected side will often have decreased output when compared to the contralateral side. This implies that functional output is impaired because of drainage impairment [14]. These changes are reinforced by histological data that demonstrated structural changes in the lacrimal gland due to the obstructive pathology. In that study, marks of ductal obstruction were indicated by increased expression of several secretory proteins (e.g., lactoferrin or lysozyme), suggesting functional changes in a glandular structure due to blocked ducts [2].

Indeed, there is a necessary component of the tear drainage mechanism called the lacrimal pump mechanism. The lacrimal pump mechanism relies on the Horner-Duverney muscle, a part of the orbicularis oculi, to create the pumping action. Contraction of the muscles combined with the blink action creates pressure on the canaliculi that works to move tears into the lacrimal sac. The muscle fibers found in the Horner-Duverney muscle are a unique composition of type II fast-twitch fibers but also type I slow-twitch fibers, which makes it a hybrid muscle that does not fatigue and can continue to perform under the frequent cycle of blinking [13].

The efficiency of tear drainage is also a function of the coordination of ocular and nasal structures, most saliently the valvular system. The rose-muller valve at the junction of the common canaliculus and lacrimal sac prevents backflow of nasal contents to the ocular surface ultimately as a unidirectional barrier [10].

Classification of nasolacrimal duct obstruction

NLDO is typically classified as either congenital or acquired, with different etiologies and clinical considerations. In neonates, the most common form is CNLDO, which is due to either incomplete canalization of the nasolacrimal duct during embryo development or membrane occlusion at the distal end of the duct, or less commonly, bony obstruction. An example of this type of obstruction is aplasia of lacrimal and salivary glands (ALSG), which has been attributed to genetic mutation of the Fgf10 gene [5]. Although CNLDO is common, many congenital cases resolve spontaneously without the need for decompression. Data from longitudinal studies revealed that up to 45% of infants past the first year of life still have CNLDO, yet they will no longer require decompression. The ultimate outcome is spontaneous resolution in the nasolacrimal duct [15].

In contrast, acquired nasolacrimal duct obstruction occurs later in life and is subclassified as either primary (PANDO) or secondary. PANDO is idiopathic and is suspected to be caused by anatomical variations, including reduction in the minimum transverse diameter of the bony nasolacrimal duct, resulting in outflow resistance [12]. Informatively, recent metagenomic studies of the lacrimal sac of the PANDO population report an unprecedented variety of microbial populations, suggesting that alterations in the local microbiota may alter inflammatory responses involved in the obstruction [16]. 

Secondary acquired NLDO may be due to several known causes. Infectious causes are not uncommon, especially with children, where organisms such as Streptococcus and Haemophilus species are identified with some frequency in cases of CNLDO with subsequent infection. Chronic inflammation is also a contributor; particularly in adults, the infiltration of immune cells in the form of T and B lymphocytes has been shown to mediate tissue remodeling and ductal narrowing in PANDO [11].

When conservative measures do not achieve the desired result, corrective surgery is the last available option. Of the various available surgical options, DCR is arguably the most effective intervention to restore tear drainage, especially in cases where the tear drainage remains chronic or complicated. The endoscopic approach to DCR has emerged as a more favored option due to the fewer number of surgical complications when using the endoscope; however, it is essential to have a good understanding of the microbiological milieu of the lacrimal sac in cases with infection, as that may aide with choice of appropriate antibiotic therapy and reduced complications post-operatively [16].

Anatomical alterations associated with NLDO

**Figure 3:**
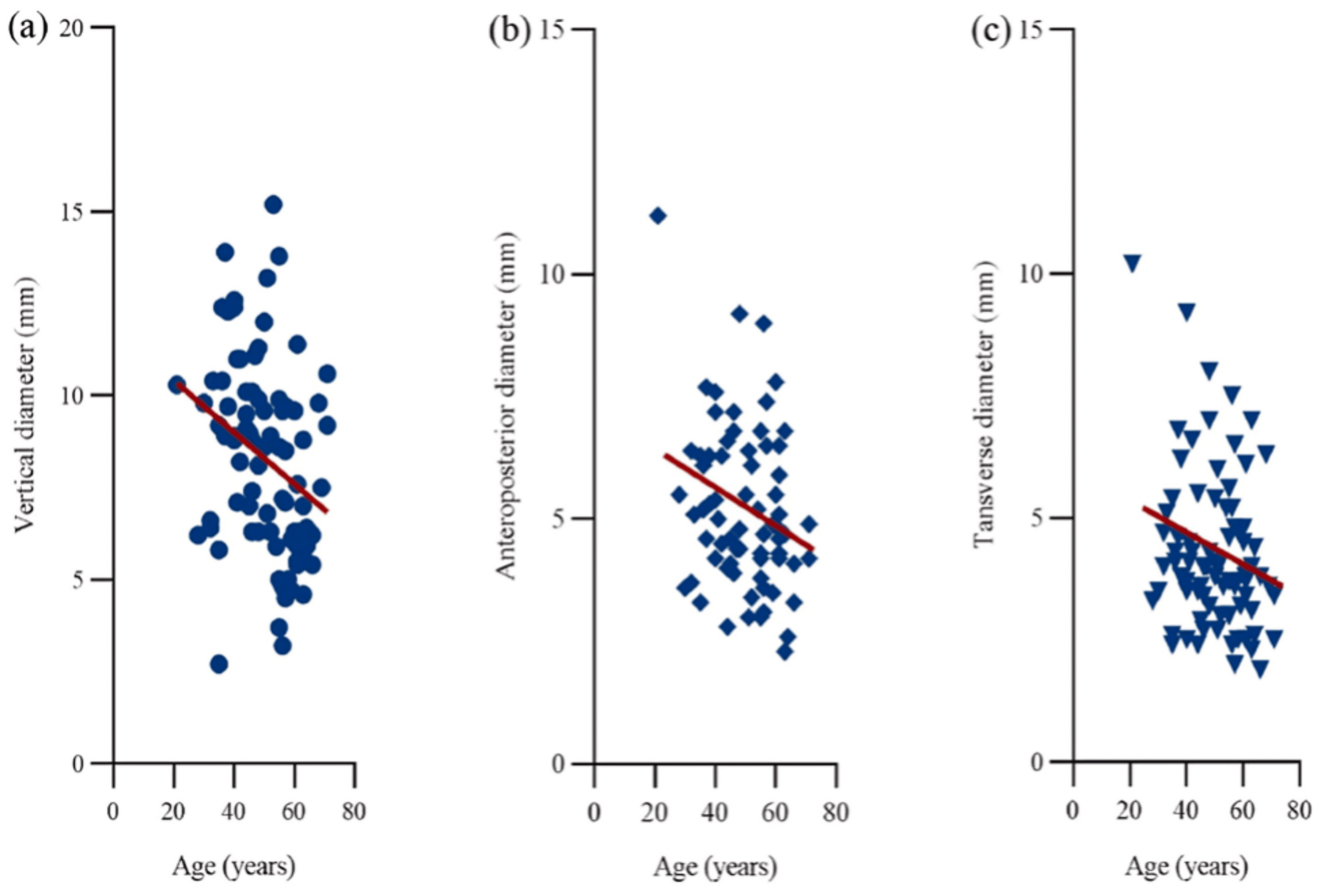
Age-related dimensional changes in the lacrimal sac. (a) Decrease in vertical diameter with aging. (b) Decline in anteroposterior diameter. (c) Reduction in transverse diameter. Scatterplots demonstrate linear negative correlations in the fully exposed group. Figure from Nie et al., licensed under CC BY-NC-ND 4.0 [17].

There are several anatomical variations that may predispose individuals to NLDO or worsen clinical manifestations. The most important of these is canalicular stenosis, canalicular stenosis is the narrowing of the canaliculi, which are small tubular structures that drain the tears from the ocular surface to the lacrimal sac through the nasolacrimal ducts. These ducts can function less effectively when narrowed, resulting in tear stagnation and epiphora. Canalicular stenosis often occurs secondary to chronic inflammation or trauma, both of which will promote fibrosis and scarring of the canalicular walls [12]. Age-related dimensional changes of the lacrimal sac, such as reductions in vertical, anteroposterior, and transverse diameters, have also been described and are illustrated in Figure 3, highlighting a potential anatomical factor in age-associated tear drainage dysfunction. 

**Figure 4:**
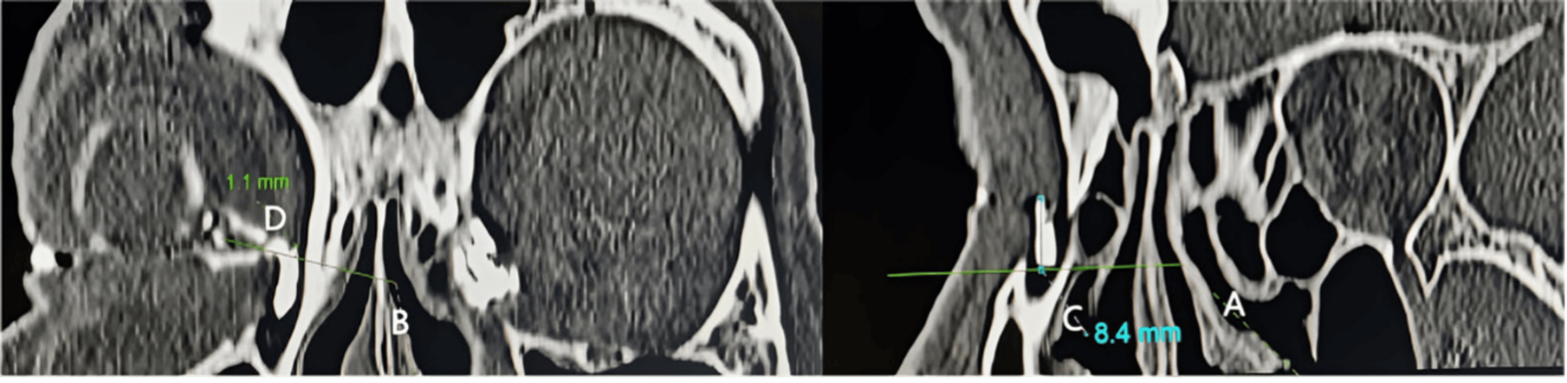
Parasagittal computed tomographic dacryocystography (CT-DCG) images showing measurements along the long axis of a small lacrimal sac. In this approach, the distance between the middle turbinate axilla (MTA) and the common canaliculus (CC) is indirectly calculated, as both structures are rarely visible simultaneously on coronal images. A vertical line (A) is drawn perpendicularly from the MTA to the long axis of the sac, while another perpendicular line (B) is drawn from the CC to the same axis. The height of the sac above the MTA (C) and above the CC (D) are then determined. The distance between the MTA and CC is obtained by subtracting the height D from height C. Figure from Yang et al., licensed under CC BY-NC-ND 4.0 [18].

Another structural cause of obstruction is the dacryocystocele, or mucocele. This is a cystic dilation of the lacrimal sac or nasolacrimal duct caused by proximal and distal obstruction, trapping fluid or mucus in the sac. Dacryocystoceles are clinically noted as a visible swelling near the medial canthus and occur in both infants and adults. Most cases noted in neonates are congenital; adult cases are most always secondary to chronic obstruction and secondary infection causing progressive distention of the sac into a mucocele [12]. Figure 4 illustrates computed tomographic dacryocystography images used to measure the dimensions of a small lacrimal sac and assess spatial relationships to adjacent nasal structures, which are relevant in preoperative planning and diagnosis [18].

In addition to variations involving the lacrimal system, some anatomical variations of the nasal system can contribute to abnormal tear drainage. Deviation of the nasal septum (DNS) is a common anatomical variant, wherein the nasal septum deviates laterally to one side, and consequently narrows the nasal passage on that side, which may compress or displace the nasolacrimal duct. Additionally, inferior turbinate hypertrophy (ITH) or enlargement of the inferior turbinate may cause further obstruction on the nasal airway, nearly always interfering with the flow of tears into the nasal cavity [19]. In this case, functional nasal breathing was interfered with, and secondary effects on the lacrimal drainage system may also develop. Surgical intervention in the form of septoplasty or turbinate reduction has been reported to effectively restore nasal airflow and alleviate NLDO symptoms in some select cases [20]. 

Further to this, nasal and sinus pathology such as chronic sinusitis, mucosal inflammation, or nasal polyps can also contribute to NLDO by obstructing the area surrounding the inferior meatus (the area of the common nasal cavity where the nasolacrimal duct drains). These lesions are either inflammatory in nature, and may spread and obstruct the tear outflow mechanism, or act as mass lesions and simply obstruct flow. In the circumstance where nothing is found on sinonasal examination, CT imaging can be useful to assess the extent of sinonasal involvement, and the potential implications for the lacrimal system let's address in turn [12,21].

Functional alterations and pathophysiology

NLDO alters the normal physiology of tear production and drainage and produces a cascade of functional changes. A major alteration is the reduction of tear flow in the affected eye compared to the intact side. The reduction in tear flow is directly related to the blockage of the tear outflow pathway such that the tears cannot efficiently drain into the nasal cavity, allowing them to pool on the ocular surface [14]. In response to impaired tear outflow or drainage, the lacrimal gland will change its function over time, and experimental studies have established a disrupted expression of secretory proteins, such as lactoferrin and lysozyme, which suggests advances by the lacrimal glands to compensate for the obstruction or adapt to the altered tear film environment [2].

Another important aspect of tear clearance is the lacrimal pump mechanism that is largely driven by the Horner-Duverney muscle, which forms part of the orbicularis oculi muscle surrounding the canaliculi. The pressure generated from the contraction of this muscle during blink, drives the tears toward the lacrimal sac. Any structural or functional alteration of this muscle (fibrosis, atrophy, and nerve damage) may affect this pumping mechanism, thereby reducing the efficiency of tear drainage even in the absence of total anatomical obstruction [22].

A further contributing aspect to the pathophysiology of NLDO is the inflammation and fibrotic remodeling of the lacrimal sac and duct. The inflammatory process, particularly in the case of PANDO, is associated with an increased expression of fibrotic markers, including TNF superfamily members LIGHT and its receptors. Furthermore, these molecular components have been associated with the degree of fibrosis and chronic inflammation in tissues affected by progressive conjunctivochalasis [23]. Additional work using single-cell RNA sequencing has identified T and B lymphocytes in the lacrimal sac, which substantiate the purported role of these immune cells mediating immune response and contributing to the fibrotic changes conferring ductal narrowing [11]. 

The involvement of microbial colonization in the clinical trajectory of NLDO might also factor into NLDO. The stasis of tears facilitates bacterial colonization, and studies have isolated varied micro-organisms in the lacrimal sac from patients with NLDO, Acinetobacter johnsonii and Escherichia coli [16]. Colonization of the lacrimal sac is actively prevalent in cases of mucinous obstruction, as the subsequent accumulation of thick secretions could have implications in bacterial colonization of the lacrimal sac [24].

Clinical presentation and diagnostic workup

**Figure 5:**
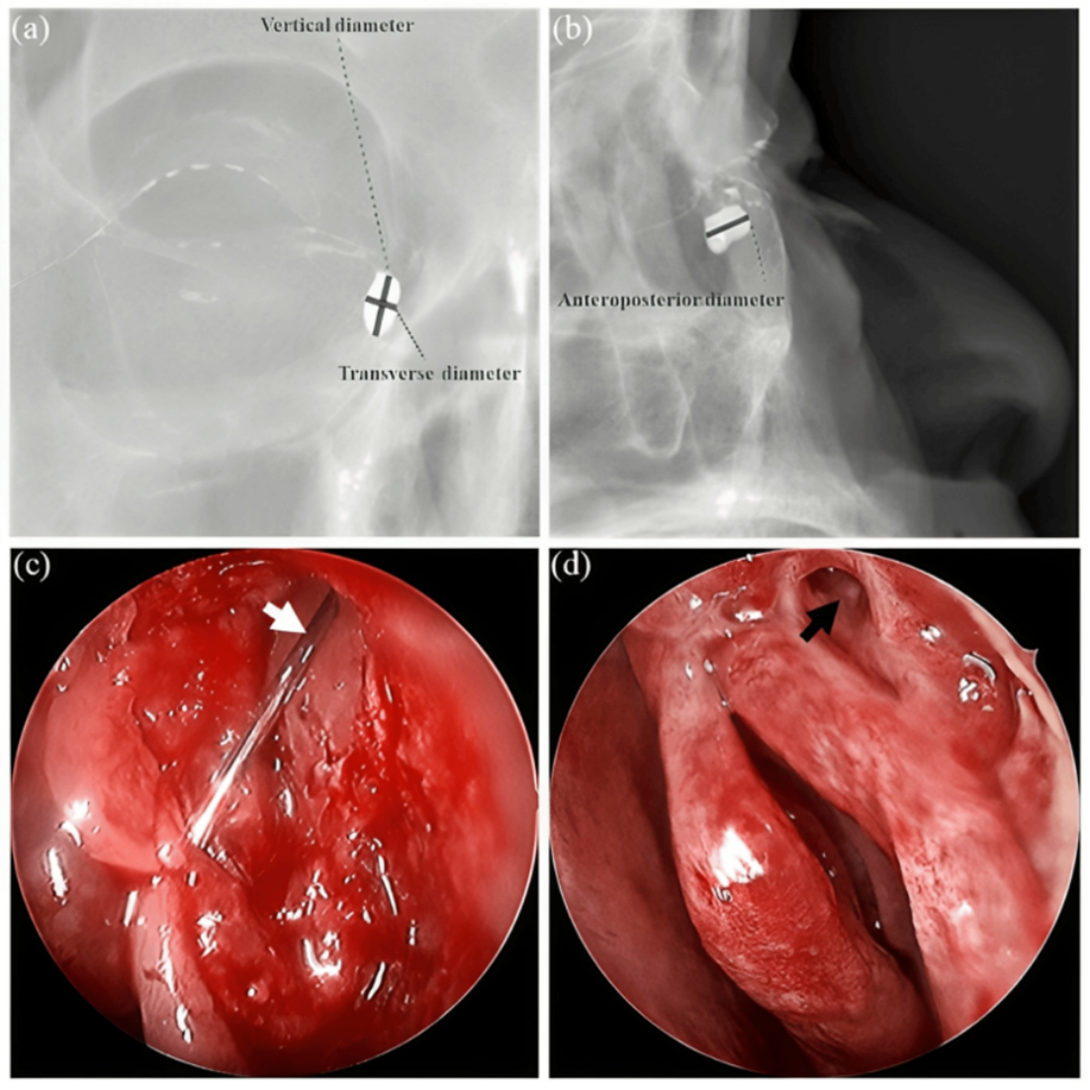
Radiographic and endoscopic findings in nasolacrimal duct obstruction (NLDO). (a) Coronal view of digital dacryocystography showing the vertical and transverse diameters of the lacrimal sac. (b) Sagittal view displaying the anteroposterior diameter. (c) Endoscopic image showing a lacrimal probe passing through the common canaliculus into the sac (white arrow). (d) Postoperative view of the ostium after endoscopic dacryocystorhinostomy (black arrow). Figure from Nie et al., licensed under CC BY-NC-ND 4.0 [17].

NLDO usually causes symptoms such as chronic epiphora, recurrent dacryocystitis, and swelling in the lacrimal sac, which are often the reasons for the patient presenting to the clinic [2,3]. The medical examination must include a complete physical examination and lacrimal irrigation to assess patency of the nasolacrimal duct and to identify if the obstruction is partial or total [3]. Imaging is key to evaluating NLDO., DCG is a particular radiography study that uses radiographic imaging to diagnose the precise location and extent of obstruction of the lacrimal drainage apparatus as shown in Figure 5 [17]. In more complicated cases, CT and magnetic resonance imaging (MRI) are useful in identifying anatomy with high-resolution imaging [17]. Alternatively, dacryoscintigraphy, which utilizes nuclear medicine imaging, is a functional measure of drainage in the lacrimal system and is often used in conjunction with DCG to provide a comprehensive evaluation [3].

Functional tests enhance the work-up process. The fluorescein dye disappearance test (FDDT) establishes how quickly dye clears from the tear film and evaluates the functional outflow of tears [25]. The Jones tests, primary and secondary, evaluate whether there is either an anatomical or functional blockage. Using fluorescein applied to the conjunctival sac in the primary Jones test indicates the nasal cavity is patent when the fluorescein is recovered, and any negative findings would involve a positive secondary Jones test which identifies functional pathology [3].

**Figure 6:**
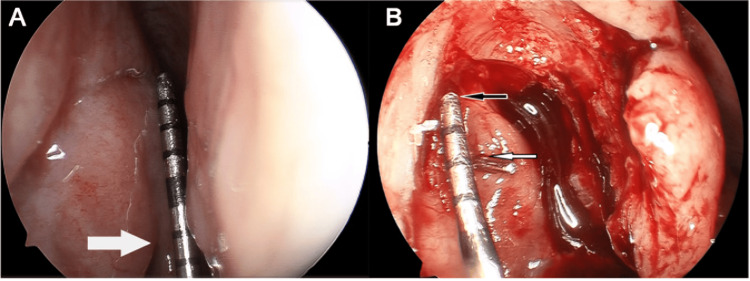
Intraoperative view of endoscopic dacryocystorhinostomy. (A) Localization of the lacrimal sac on the lateral nasal wall using a graduated probe; the middle turbinate axilla is indicated by the white arrow. (B) Exposure of the sac superior fundus (black arrow) and the opening of the common canaliculus (white arrow). Figure reproduced from Yang et al., licensed under CC BY 4.0 [18].

Endoscopic DCR is often performed to bypass the blocked segment and create a new outflow into the nasal cavity. Figure 6 illustrates an intraoperative view of this procedure, highlighting anatomical references such as the middle turbinate axilla, the lacrimal sac fundus, and the canaliculus. Endoscopic DCR has been shown to be a reliable method of reconnection in total obstruction and consistently has a high success rate [3]. Lacrimal intubation preserves patency by placing a silicone stent through the nasolacrimal duct and is an intervention often restricted to functional obstruction with a varied success rate based on patient factors and surgical technique [3,26].

Therapeutic approaches

Conservative management is the first line of therapy for CNLDO, because it has a high occurrence of spontaneous resolution. Current literature reports spontaneous resolution rates for CNLDO to be as high as 96%, and this is largely attributed to certain conservative methods including lacrimal sac massage and institution of topical antibiotic eye drops [1]. Furthermore, cases in infants were not the only instances of spontaneous resolution as many cases continued to experience spontaneous resolution beyond the first year of age, with one report indicating spontaneous resolution by approximately 17.8 months of age [14]. Medical therapy plays a vital supportive role in the conservative approach. One potential advantage is the use of topical antibiotics, which are often used to treat secondary bacterial infections associated with ongoing ductal obstruction [1].

When conservative measures fail, surgery is required. Probing and irrigation are the most commonly performed first-line surgical procedures. Probing is associated with high success rates, especially with performance of the procedure under dacryoendoscopy, as it improves visualization and facilitates performance of the procedure [17,27]. Age also plays a role; infants aged 12 to 24 months typically have great success rates, while older children typically have better outcomes with adjunctive or alternative procedures [26]. Balloon dacryoplasty is a new and minimally invasive alternative to probing and irrigation, with several recently published studies showing excellent outcomes in patients who had previously failed probing and irrigation. The procedure is performing well in part due to the high rates of success and low complication rate, therefore surgeons are more prone to perform the surgery in selected patients [28].

In more complicated cases or when anatomical variabilities are present, DCR is indicated. The external approach is the accepted gold standard and was particularly effective when structural abnormalities exist, but it is also the most invasive method [28]. Endoscopic DCR is less invasive and has comparable success rates to the external procedure, with equivalent successful rates of complete nasolacrimal duct obstruction [3]. Supplementary techniques, including silicone stent insertion or preservation of a mucosal flap, may improve functional outcomes but usually require their own consideration [29]. Correcting any anatomical defect has a major impact on improving the outcome. When a deviation of the septum contributes to the obstruction of the nasolacrimal system, septoplasty should be completed during the procedure. Possible concurrent turbinate reduction, or inferior turbinate fracture, may be performed with probing and in elderly pediatric patients to improve access and surgical experience for an improved long-term outcome [27,29]. 

Prognosis and outcomes

Prognostic factors are important for determining success in intervention for NLDO. The most significant prognostic factor is age, and younger patients will most often have success rates of a much higher proportion, especially pediatric patients under 36 months with silicone tube intubation. Several studies have achieved a 100% success rate within this age group [23]. In contrast, older age is associated with lower surgical effect and treatment success, for example with endoscopic dacryocystorhinostomy (eDCR), which not only had less success, but less success over time as older age decreased efficacy [30]. 

The obstruction type will also yield variations in success, as structural defects would be better identified as having membranous obstruction at the valve of Hasner illuminated through some form of dacryoendoscopy, improving precision of diagnosis and selection of good treatment options [22]. There are also variations in each surgical modality with respect to the success and complication statistics. For example, dacryoendoscopy-assisted intubation or trans canalicular microdrill dacryoplasty (MDP) both proved successful, and more recently, MDP has been an advanced minimally invasive approach that has an important consequence with low risk [23,28].

Yet, recurrence continues to be an ongoing problem. Long-term follow-up has demonstrated that procedures like eDCR have declining success rates over time, with success rates falling from 92.5% immediately following surgery to 80% at 10 years [30]. Although complications are usually infrequent across all techniques, intraoperative discomfort and postoperative pain can be affected by surgical time and total laser energy. For MDP, significant bleeding is infrequent, ultimately providing more safety [28]. When measuring efficacy from a therapy standpoint, functional outcomes and quality of life are both important measures to consider. Functional success is defined by a patient-reported bothering but can vary greatly. For transcanalicular laser dacryocystorhinostomy, functional success has been shown to be only 33.81% three years later [25]. Patient satisfaction is also quite variable; patients who received MDP report a moderate mean satisfaction of 6.9 out of 10. For this reason, complete assessments of objective and subjective outcomes should be included in treatment planning [28].

## Conclusions

Anatomical and functional changes of the nasolacrimal system are important factors that play a significant role in the development of NLDO. A comprehensive understanding of these structures, along with their own unique anatomical variations, drainage mechanisms, and possible inflammatory characteristics, will produce accurate diagnoses and more effective therapeutic decision-making.

The management of NLDO requires an individualized approach based on the patient's age, possible cause, and the individual anatomy surrounding it. Treatment options range from conservative to surgical. Additional surgical procedures to improve overall recovery can also be considered, such as septoplasty or dacryoendoscopy, which facilitate both the clinical and functional recovery of patients.
